# DMT1 Protects Macrophages from *Salmonella* Infection by Controlling Cellular Iron Turnover and Lipocalin 2 Expression

**DOI:** 10.3390/ijms23126789

**Published:** 2022-06-17

**Authors:** Manuel Grander, Alexander Hoffmann, Markus Seifert, Egon Demetz, Philipp Grubwieser, Christa Pfeifhofer-Obermair, David Haschka, Günter Weiss

**Affiliations:** 1 Department of Internal Medicine II, Medical University of Innsbruck, 6020 Innsbruck, Austria; manuel.grander@student.i-med.ac.at (M.G.); alexander.hoffmann@i-med.ac.at (A.H.); markus.seifert@i-med.ac.at (M.S.); egon.demetz@i-med.ac.at (E.D.); philipp.grubwieser@i-med.ac.at (P.G.); christa.pfeifhofer@i-med.ac.at (C.P.-O.); 2 Christian Doppler Laboratory for Iron Metabolism and Anemia Research, Medical University of Innsbruck, 6020 Innsbruck, Austria

**Keywords:** nutritional immunity, iron, DMT1, lipocalin 2, *Salmonella* Typhimurium, macrophage

## Abstract

Macrophages are at the center of innate pathogen control and iron recycling. Divalent metal transporter 1 (DMT1) is essential for the uptake of non-transferrin-bound iron (NTBI) into macrophages and for the transfer of transferrin-bound iron from the endosome to the cytoplasm. As the control of cellular iron trafficking is central for the control of infection with siderophilic pathogens such as *Salmonella* Typhimurium, a Gram-negative bacterium residing within the phagosome of macrophages, we examined the potential role of DMT1 for infection control. Bone marrow derived macrophages lacking DMT1 (DMT1fl/fl*^LysMCre^*^(+)^) present with reduced NTBI uptake and reduced levels of the iron storage protein ferritin, the iron exporter ferroportin and, surprisingly, of the iron uptake protein transferrin receptor. Further, DMT1-deficient macrophages have an impaired control of *Salmonella* Typhimurium infection, paralleled by reduced levels of the peptide lipocalin-2 (LCN2). LCN2 exerts anti-bacterial activity upon binding of microbial siderophores but also facilitates systemic and cellular hypoferremia. Remarkably, nifedipine, a pharmacological DMT1 activator, stimulates LCN2 expression in RAW264.7 macrophages, confirming its DMT1-dependent regulation. In addition, the absence of DMT1 increases the availability of iron for *Salmonella* upon infection and leads to increased bacterial proliferation and persistence within macrophages. Accordingly, mice harboring a macrophage-selective DMT1 disruption demonstrate reduced survival following *Salmonella* infection. This study highlights the importance of DMT1 in nutritional immunity and the significance of iron delivery for the control of infection with siderophilic bacteria.

## 1. Introduction

Nutritional immunity states that host resistance is dependent on its ability to restrict access of essential nutrients to microbes [1,2,3]. While iron is, on the one hand, crucial for mammalian physiology by promoting erythropoiesis, metabolic functions and cellular respiration [4,5], the metal is also involved in the immune response by exerting subtle effects on cytokine formation and immune cell differentiation [6]. On the other hand, sufficient access to iron determines microbe proliferation and pathogenicity [7]. Consequently, iron disorders, as displayed in hereditary hemochromatosis or thalassemia, affect the susceptibility of host cells to infection. Therefore, in response to invading pathogens, immune defense mechanisms aim at restricting iron availability, ultimately resulting in the development of anemia of chronic disease [8,9]. Macrophages are at the center of innate and nutritional immunity. Tissue macrophages in the liver and spleen recycle and deliver approximately 90–95% of the body’s daily need for iron by phagocytosing senescent red blood cells. In addition, it is evident that macrophages are critical for innate immunity by engulfing invaders and initializing the inflammatory immune response. Sepsis thus confronts myeloid cells with a dilemma: they are vital in fighting pathogens but simultaneously responsible for upholding iron homeostasis [10].

Therefore, macrophages regulate essential iron transfer pathways to limit iron availability to microbes. Transferrin-bound iron (TBI) uptake is diminished in response to intracellular infection [11]. Notably, microbes such as *Francisella tularensis* or *Mycobacterium avium* manipulate the transferrin cycle to gain access to iron [12]. Conversely, pleiotropic effects of transferrin regulation become evident as macrophage HFE disruption, a mutation pathognomonic for hereditary hemochromatosis that affects TBI uptake, is associated with resistance to *Salmonella* infection [13]. Another defense mechanism to restrict iron access to pathogens is linked to the expression of the anti-microbial peptide lipocalin 2 (LCN2). This antimicrobial peptide binds bacterial iron scavenger proteins, particularly promoting resistance to enterobactin-producing bacteria [14]. Of note, also endogenous catechols, such as 2,5-Dihydroxybenzoic acid or the recently examined dopamine, can serve as xeno-siderophores, thereby affecting bacterial proliferation [15,16]. Further, the expression of ferritin (FRT), an intracellular iron storage protein, reduces the labile iron pool (LIP) in macrophages, which renders it inaccessible to pathogens [17]. This is particularly important, as low LIP appears to induce crucial transcription factors such as hypoxia-inducible factor 1a (HIF1a) and nuclear factor interleukin 6, which transactivate anti-microbial immune effector pathways [18].

Divalent Metal Transporter 1 (DMT1) is involved in at least two crucial pathways of iron homeostasis in macrophages. Those are the import of non-transferrin-bound iron (NTBI) from the extracellular space and the export of iron originating from TBI from the early endosome to the cytoplasm [19]. Previously, we demonstrated that pharmacological induction of DMT1 via nifedipine reverses primary and secondary iron overload by promoting iron mobilization from the spleen and liver [20]. Furthermore, in vitro and in vivo experiments demonstrated that DMT1 activation by nifedipine mediates host resistance to *Salmonella* infection, which could be partly traced back to consecutive induction of the iron exporter ferroportin-1 (FPN1), and thus, limitation of intracellular iron access for bacteria [21]. To study the impact of DMT1 on host control of infections, we used the Cre/Lox technology for selective disruption of DMT1 in the myeloid cell line [22]. Mice harboring a constitutive disruption of DMT1, as observed in the microcytic anemia mouse or “Belgrad rat“, developed severe anemia and bared increased perinatal lethality, indicating essential functions of DMT1 [23,24]. Interestingly, DMT1^*LysMCre*(+)^ mice are vital and develop normally. This study reveals that iron homeostasis in bone marrow-derived macrophages (BMDMs) is critically controlled by DMT1. Moreover, it demonstrates the importance of DMT1 in innate immunity for pathogen control.

## 2. Results

### 2.1. DMT1 Alters Key Molecules of Iron Homeostasis

**Figure 1:**
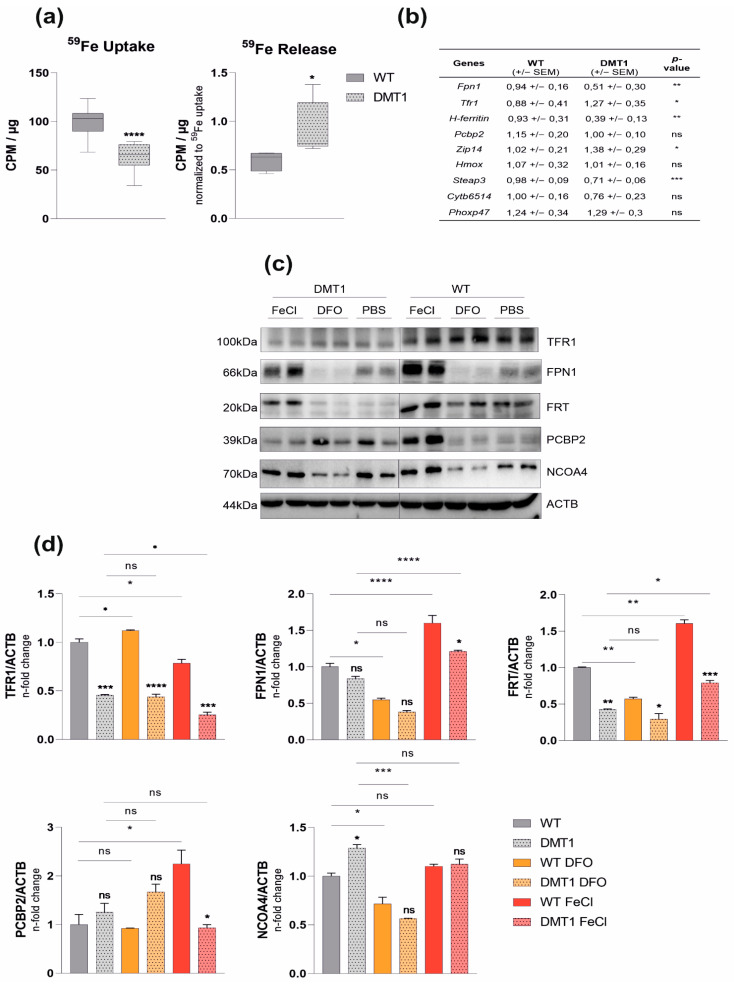
**Role of DMT1 in bone-marrow-derived macrophages (BMDMs):** (**a**) Uptake (*n* = 4) and release assay (*n* = 2): BMDMs were stimulated with radioactive ^59^Fe-citrate, and uptake/release was measured with a gamma-counter (counts per minute—cpm) and normalized to the protein concentration (µg). ^59^Fe release was normalized to uptake data; (**b**) Relative mRNA expression (*n* = 5) of genes involved in iron transport: Ribosomal Protein L4 (Rpl4) was used as a reference gene (averages ± SEM); (**c**) BMDMs (*n* = 2) were stimulated with 50 µM FerricChloride (FeCl) or Deferoxamine (DFO) overnight. Protein levels of transferrin receptor 1 (TFR1), ferroportin (FPN1), ferritin (FRT), Poly(rC)-binding protein 2 (PCBP2), Nuclear Receptor Coactivator 4 (NCOA4) and βActin(ACTB); (**d**) Densitometric quantification of Western blot results; see Figure S5 for a schematic overview of the discussed proteins. Data were compared by a two-tailed unpaired *t*-test (two groups) or analysis of variance (ANOVA) using the Bonferroni correction (more than two groups); *n* = mice per group; Data are expressed as box plots showing whiskers with minimum to maximum. Statistical significance: * *p* < 0.05, ** *p* < 0.01, *** *p* < 0.001, **** *p* < 0.0001, ns, no significance of differences; DMT1: DMT1 fl/fl*^LysMCre^*^(+)^, WT: wildtype.

Mice with homozygously floxed DMT1 alleles (DMT1 fl/fl) were bred to littermates with Lysozyme 2-dependent Cre-Recombinase (LysMCre Cre+), thus selectively targeting DMT1 disruption in myeloid cells, including macrophages and neutrophils. The ablation of DMT1 was confirmed by genotyping and immunoblot analysis (Figure S1a,b). DMT1fl/fl*^LysMCre^*^(+)^ mice bred normally when compared with their littermates bearing functional DMT1 in macrophages (DMT1fl/fl*^LysMCre^*^(−)^). Importantly, DMT1fl/fl*^LysMCre^*^(+)^ and wildtype mice presented with comparable weight and normal blood counts at the age of eight to 12 weeks (Figure S2). While BMDMs of DMT1fl/fl*^LysMCre^*^(+)^ mice showed reduced NTBI uptake, as evidenced by diminished ^59^Fe-Citrate uptake (Figure 1a), subsequent release of ^59^Fe was increased upon loss of DMT1 (Figure 1a). In addition, mRNA levels of the alternative iron importer *Zip14* were elevated, indicating that upregulation is not sufficient to compensate for reduced NTBI uptake upon loss of DMT1 (Figure 1b). Conversely, the levels of ferrireductase *Steap3* mRNA were downregulated upon the disruption of DMT1, suggesting reduced TBI export from the endosome (Figure 1b). Analyzing the correspondent expression of *Tfr1,* we found increased mRNA levels being indicative for intracellular iron deficiency (Figure 1b). This was in line with reduced FRT levels and elevated expression of the FRT-degrading molecule Nuclear Receptor Coactivator 4 (NCOA4) in DMT1-deficient macrophages, although at the investigated time point, TFR1 protein levels were also low (Figure 1b–d). Of note, while *Fpn1* mRNA expression was reduced in DMT1-deficient macrophages this did not result in altered protein levels, as compared to BMDMs with functional DMT1, also pointing to the importance of different regulatory mechanisms for the control of mRNA and protein expression of TFR1 and FPN1 [5].

In addition, the response of DMT1-deficient macrophages to iron challenge was evaluated. As expected, ferric chloride (FeCl3) stimulation upregulates FRT and FPN1, but suppresses TFR1 expression in DMT1 fl/fl^*LysMCre*(−)^ BMDMs (Figure 1c,d). Conversely, iron chelation by deferoxamine (DFO) augments TFR1 but decreases FPN1 and FRT expression. DMT1fl/fl*^LysMCre^*^(+)^ macrophages display attenuated or no significant response to iron fluctuations. FPN1 and FRT upregulation is diminished in DMT1fl/fl*^LysMCre^*^(+)^ upon iron stimulation. Of note, while iron availability affects the expression of the intracellular iron chaperone poly(rC)-binding protein 2 (PCBP2), DMT1 depletion results in increased PCBP2 levels, which are not affected by iron challenge.

### 2.2. DMT1 Mediates Macrophage Resistance to Salmonella Infection

**Figure 2:**
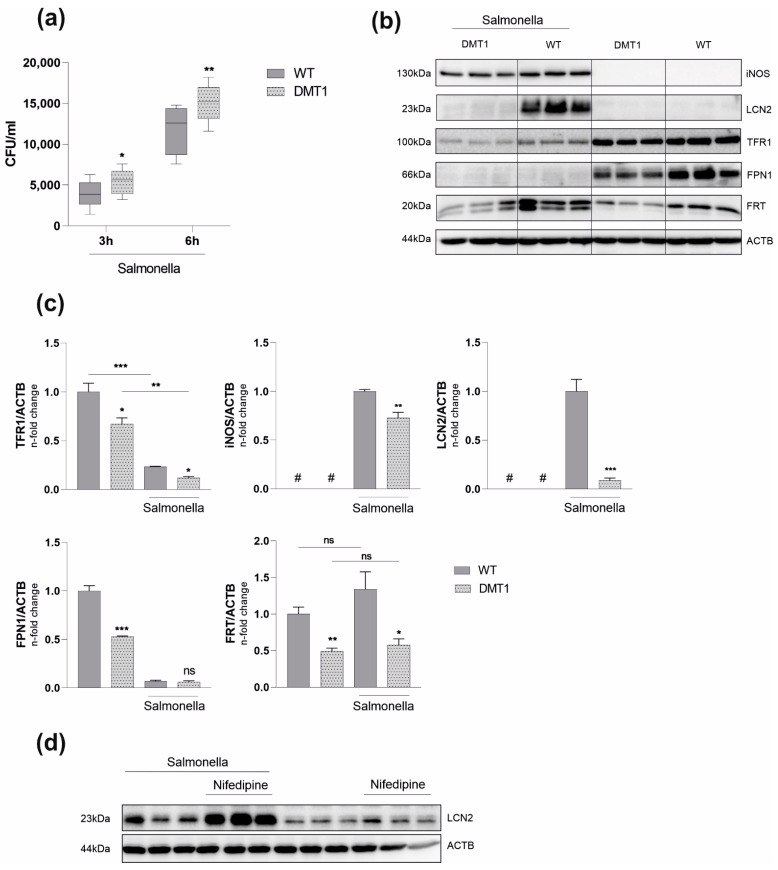
**DMT1 protects BMDMs against *Salmonella* infection.** (**a**) Colony forming units (CFU) of *Salmonella* after 3 and 6 h respectively (*n* = 5); (**b**) Protein levels of inducible nitric oxide synthase (iNOS), lipocalin-2 (LCN2), transferrin receptor 1 (TFR1), ferroportin (FPN1), FRT (FRT) and βActin (ACTB) 6 h post infection (*n* = 3). (**c**) Densitometric quantification of Western blot results; (**d**) RAW264.7 macrophages were stimulated with 50 µM Nifedipine for 24 h. The protein level of LCN2 was determined via Western Blot analysis; Duplicates or triplicates from at least two independent experiments were compared by a two-tailed unpaired *t*-test (two groups) or analysis of variance (ANOVA) using a Bonferroni correction (more than two groups); *n* = mice per group. Error bars expressed as SEM. Box plots display whiskers with the minimum to maximum. Statistical significance * *p* < 0.05, ** *p* < 0.01, *** *p* < 0.001, ns, no significance of differences; #, below detection level; DMT1: DMT1 fl/fl*^LysMCre^*^(+)^, WT: wildtype.

**Figure 3:**
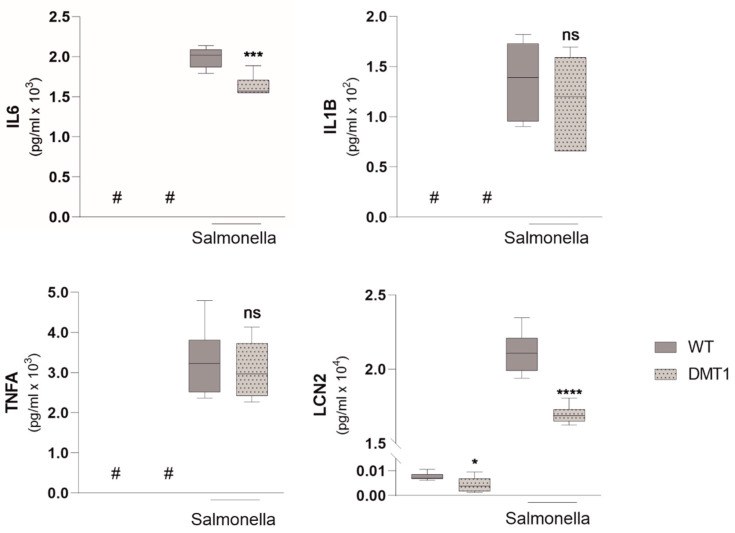
**DMT1 modulates Interleukin 6 and Lipocalin 2 formation.** Enzyme linked immunosorbent assay (ELISA) from cell culture supernatants for Interleukin 6 (IL-6), Interleukin 1b (IL-1b), Tumor-necrosis factor alpha (TNFa), Lipocalin 2 (LCN2) (*n* = 2); Duplicates or triplicates from at least two independent experiments were compared by a two-tailed unpaired *t*-test (two groups) or analysis of variance (ANOVA) using Bonferroni correction (more than two groups); *n* = mice per group. Box plots display whiskers with the minimum to maximum. Statistical significance * *p* < 0.05, *** *p* < 0.001, **** *p* < 0.0001, ns, no significance of differences; #, below detection level; DMT1: DMT1 fl/fl*^LysMCre^*^(+)^, WT: wildtype.

Macrophage iron retention, as reflected by increased FRT expression and higher intracellular iron content, is commonly observed in response to inflammation [8,9,25]. Intracellular pathogens are able to exploit these retained iron pools in macrophages, thereby promoting proliferation and pathogenicity [26,27]. Importantly, inducing iron retention by defined inflammatory stimuli did not mitigate the observed phenotype in DMT1-deficient macrophages. Though elevated in both genotypes, TFR1 and FRT were still reduced compared to DMT1 fl/fl^*LysMCre*(−)^ BMDMs (Figure S3a,b). As the regulation of iron metabolism proteins was indicative for a low cytoplasmatic iron milieu (Figure 1c,d) we hypothesized a protective role of DMT1 upon infection with intracellular bacteria. To test our assumption, we infected BMDMs with the siderophilic, intracellular bacterium *Salmonella serovar* enterica Typhimurium (*Salmonella*). Unexpectedly, the absence of DMT1 caused a significantly higher *Salmonella* burden at three- and six-hours post-infection (Figure 2a). Nonetheless, *Salmonella* infection did not alter the divergent regulation of key iron molecules between both genotypes (Figure 2b–d). In response to *Salmonella*, TFR1 was downregulated as compared to uninfected cells; however, DMT1-deficient macrophages still displayed lower TFR1 levels compared to wild-type macrophages (Figure 2b–d). FRT expression was reduced upon loss of DMT1 under control conditions and upon infection; however, infection resulted in the expression of FRT in both genotypes. Of interest, at this early time point post-infection, the protein expression of the iron exporter FPN1 was diminished in both genotypes, although bacterial numbers were significantly lower in BMDMs bearing functional DMT1 (Figure 2a–c). Therefore, we focused on possible alternative explanations for differences in the *Salmonella* burden between the two genotypes. The protein expression of inducible nitric oxide synthase (iNOS) was slightly reduced in DMT1-deficient cells despite an increased *Salmonella* burden. Strikingly, we found greatly reduced intracellular LCN2 levels in infected DMT1fl/fl*^LysMCre^*^(+)^ BMDMs (Figure 2b–d). Furthermore, analysis of extracellular immune mediators confirmed decreased LCN2 levels in supernatants upon loss of DMT1 (Figure 3). To confirm the hypothesis that DMT1 stimulates LCN2 expression, RAW 264.7 macrophages were stimulated with nifedipine, a well-known pharmacological DMT1 activator. Importantly, nifedipine significantly increased LCN2 expression upon *Salmonella* infection as compared to macrophages treated with *Salmonella* alone.

Additionally, iron and inflammatory stimuli were both robust inducers of LCN2 in wildtypes, but we only found attenuated induction of LCN2 in DMT1-deficient macrophages (Figure S4). When analyzing for other immune mediators in cell culture supernatants, we found slightly reduced interleukin-6 (IL6) but unaltered interleukin-1ß (IL1ß) and tumor necrosis factor α (TNFA) formation in infected DMT1-deficient BMDMs, as compared to wildtype BMDMs (Figure 3).

### 2.3. DMT1 Modulates Iron Availability in Control and Infected Macrophages

**Figure 4:**
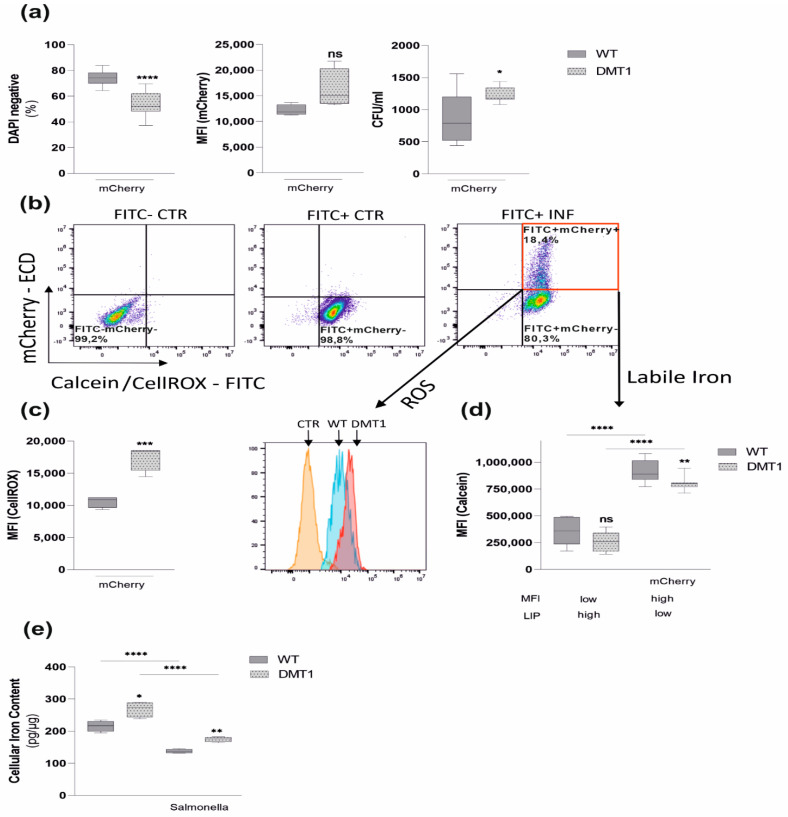
**DMT1 modulates iron availability in control and infected macrophages.** Macrophages were infected with red fluorescent protein expressing *Salmonella* strains (*mCherry*) for 6 h. (**a**) The percentage of DAPI-negative cells indicating macrophage viability (*n* = 3) and mean fluorescence intensity (MFI) of *mCherry* infected macrophages (*n* = 3) suggesting *mCherry* proliferation. (**b**) Samples were stained either with CellROX Green (**c**) to determine oxidative stress or Calcein-AM (**d**) to measure LIP. A representative experiment with CellROX is shown using the following template: unstained control (CTR, FITC-*mCherry*−), stained control (FITC+*mCherry*−), stained infected (INF, FITC+*mCherry*+) BMDMs. (**c**) Reactive oxygen species (ROS) among *mCherry* (FITC+*mCherry*+)-containing macrophages were determined by flow cytometry. Data from 3 experiments are shown. Representative histogram including the FITC+*mCherry*-control normalized to mode is shown. (**d**) Calcein quench experiments were performed: DMT1 decreased the labile iron pool in infected macrophages (FITC+*mCherry*+) and uninfected macrophages (FITC+*mCherry*−), as evidenced by increasing Calcein-mediated mean fluorescence intensity (MFI). (**e**) Total iron content of control and infected macrophages was measured by atomic absorption (*n* = 2); Duplicates or triplicates from at least two independent experiments were compared by two-tailed unpaired *t*-test (two groups) or analysis of variance (ANOVA) using Bonferroni correction (more than two groups); *n* = mice per group. Box plots display whiskers with the minimum to maximum. Statistical significance * *p* < 0.05, ** *p* < 0.01, *** *p* < 0.001, **** *p* < 0.0001, ns, no significance of differences; DMT1: DMT1 fl/fl*^LysMCre^*^(+)^, WT: wildtype, *mCherry*: red fluorescent protein (RFP)-expressing *Salmonella*.

As LCN2 critically affects iron homeostasis in infected macrophages [28], we attempted to better define the pathophysiological consequences of reduced LCN2 production in DMT1fl/fl*^LysMCre^*^(+)^ macrophages. Thus, BMDMs were infected with *Salmonella* expressing red-fluorescent protein (*mCherry*). Although no difference in MFI of *mCherry* was observed, the correspondent CFUs indicated a significantly increased bacterial burden upon DMT1 disruption (Figure 4a). Intriguingly, upon infection with *mCherry*, increased cell death (Figure 4a) combined with elevated levels of reactive oxygen species (ROS) was observed in DMT1fl/fl*^LysMCre^*^(+)^ macrophages (Figure 4b,c). A crucial catalyst for ROS formation via Fenton reaction is the availability of redox-active “free iron”. Upon *mCherry* infection DMT1-deficient macrophages, as well as wild-type macrophages, reduced their labile iron pool (LIP). However, DMT1-deficient macrophages still displayed significantly elevated levels of their LIP (Figure 4b,d), which might become available for intra-macrophage bacteria, thereby promoting their survival and proliferation. In our infection model, FPN1, the sole known iron exporter, is strongly reduced upon *Salmonella* infection (Figure 2b–d). As extracellular LCN2 is strongly expressed upon infection with *Salmonella* (Figure 3), LCN2 expression is associated with reduced macrophage total iron content, which is based on previous observations in hemochromatosis mice [29]. Strikingly, though infection reduces total iron content in both genotypes, the loss of DMT1 significantly increases intracellular iron content in uninfected and infected macrophages in comparison to wildtype macrophages (Figure 4e). These results confirm an impaired control of iron trafficking in DMT1fl/fl*^LysMCre^*^(+)^ macrophages upon infection. This may be based on the DMT1-mediated reduction of LCN2 expression and subsequently diminished LCN2-mediated cellular iron export.

### 2.4. Macrophage DMT1 Mediates Resistance to Salmonella In Vivo

**Figure 5:**
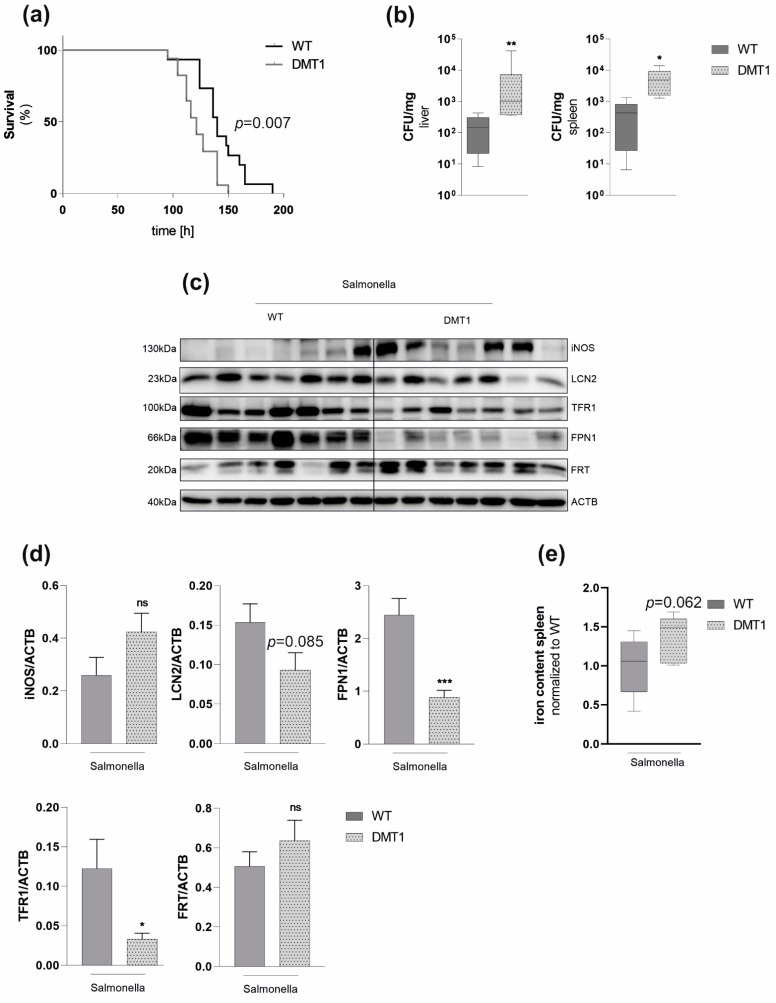
**Macrophage DMT1 protects against *Salmonella* infection in vivo** (**a**) Mice were subjected to a lethal dose of *Salmonella* by injecting 500 colony-forming units (CFU) intraperitoneally (*n* = 15). Survival data between control and mutant mice were compared using Cox regression and the Kaplan–Meier method using Wilcoxon’s test. (**b**) CFU normalized to the weight of spleen or liver from mice infected with *Salmonella* after 72 h (*n* = 7) (**c**) Western Blot for selected genes from spleens 72 h after infections (*n* = 7), (**d**) Densitometric quantification of the Western Blot, (**e**) Tissue iron of the spleen normalized to protein and wildtype control (*n* = 7). Duplicates or triplicates from at least two independent experiments were compared by a two-tailed unpaired *t*-test (two groups) or analysis of variance (ANOVA) using a Bonferroni correction (more than two groups); *n* = mice per group. Error bars express the SEM. Box plots display whiskers with the. minimum to maximum. Statistical significance * *p* < 0.05, ** *p* < 0.01, *** *p* < 0.001, ns, no significance of differences; DMT1: DMT1 fl/fl*^LysMCre^*^(+)^, WT: wildtype.

We then were interested in whether macrophage DMT1 also protects mice against infection with *Salmonella*. We thus exposed mice to a lethal dose of *Salmonella*. As expected, DMT1fl/fl*^LysMCre^*^(+)^ succumbed to death earlier than control littermates (Figure 5a). Furthermore, increased vulnerability to *Salmonella* was paralleled with a higher bacterial burden in the liver and spleen at 72 h after infection (Figure 5b). The analysis of protein expression in tissue samples at that time revealed reduced expression of LCN2 in infected DMT1fl/fl*^LysMCre^*^(+)^ mice, which, however, did not reach statistical significance (Figure 5c,d). This indicates that alternative non-myeloid cells at least partly compensated diminished splenic LCN2 levels in DMT1fl/fl*^LysMCre^*^(+)^ macrophages, as already described previously for hepatocyte selective LCN2 disruption in the liver [30]. Comparable to our in vitro results, TFR1 was significantly downregulated in spleens of DMT1fl/fl*^LysMCre^*^(+)^ mice in comparison to wild-type mice. Intriguingly, splenic FPN1 expression was significantly lower in mice with myeloid DMT1 disruption than wildtype littermates (Figure 5c,d). In parallel with reduced TFR1 and FPN1 expression, we observed higher FRT levels in DMT1fl/fl*^LysMCre^*^(+)^, which are all indicative for increased cellular iron retention in the spleen. To test this hypothesis, we determined tissue iron of the correspondent spleens and found a strong tendency towards increased iron content upon loss of DMT1 (Figure 5e). This is also in line with our in vitro data and, thus, with a condition favoring *Salmonella* proliferation.

## 3. Discussion

Macrophages hold a key position in nutritional immunity by limiting bacterial iron access. Of note, cellular iron homeostasis is regulated differently in macrophages in response to the challenge with intra- and extracellular bacteria. This includes the induction of distinct iron trafficking avenues upon infection and subsequent activations of anti-microbial immune effector pathways [31,32].

Key regulatory molecules of cellular iron metabolism include the iron exporter FPN1 and the siderophore binding peptide LCN2 but also other innate immune genes which affect iron homeostasis, such as lactoferrin, calprotectin, nitric oxide, natural resistance associated macrophage protein-1 and transferrin, which all influence bacterial iron access [33,34,35]. Recently, the Iron Regulatory Protein 2, which orchestrates intracellular iron homeostasis and the iron storage protein H-FRT, has been recognized as being essential for host resistance to infection with intracellular bacteria [17,36]. To the best of our knowledge, no data on the role of DMT1 in macrophage-mediated control of infection was available. Therefore, we used DMT1fl/fl*^LysMCre^*^(+)^ mice, which were raised and bred normally. As macrophages are critical to supplying the erythroid niche with iron, this has been to some extent surprising to us, indicating that C57BL/6J mice are able to compensate for myeloid disruption of DMT1. Moreover, the mosaic activity of Lysozyme 2, and consequently, incomplete disruption of DMT1, may attenuate the observed phenotype [37]. In our in vivo experiments, we could not only recapitulate a higher bacterial load in mice with myeloid DMT1 deficiency, but we even observed a reduced survival. This can be traced back to nutritional immunity, as cellular iron homeostasis as well as bacterial iron delivery is modified as a result of DMT1 absence. Our in vivo results indicated reduced FPN1 induction in DMT1fl/fl*^LysMCre^*^(+)^ mice and thus suggested increased iron access for intracellular bacteria. This is in line with data regarding the DMT1 inducer nifedipine which demonstrates improved infection control via DMT1-mediated FPN1 induction and subsequent reduction of bacterial iron access [21]. In contrast to a previous report that found increased renal LCN2 expression upon constitutive DMT1 disruption [38], we uncovered a positive impact of DMT1 functionality towards the expression of the bacterial siderophore scavenging peptide LCN2 in macrophages. This is linked to host control of *Salmonella* infection, as we could demonstrate herein that DMT1 induction by nifedipine increases LCN2 expression and that nifedipine-mediated LCN2 induction in RAW267.4 macrophages reduces intramacrophage *Salmonella* multiplication [39]. Interestingly, our data reveal that LCN2 deficiency is functionally associated with an increased LIP. Our studies reveal that iron stimulation alone is sufficient to induce LCN2 in WT*^LysMCre^*^(−)^ but not in DMT1fl/fl*^LysMCre^*^(+)^ BMDMs, suggesting an additional role for LCN2 in physiological iron homeostasis (Figure S3). Previous data have suggested that LCN2 is able to export iron from macrophages [29,40]. Similarly, we observed increased cellular iron content and reduced LCN2 expression in DMT1-deficient macrophages, suggesting that LCN2 effectively reduces total iron content by export most likely independent of the PCBP2-FPN1 axis [41]. Infection also resulted in reduced LCN2 induction in DMT1fl/fl*^LysMCre^*^(+)^ macrophages, which was accompanied by an increased intracellular LIP and higher intracellular bacterial burden.

However, the analysis of spleen samples from infected mice revealed no significant differences in LCN2 expression, which is likely explained by the fact that LCN2 is also produced in large quantities by epithelia [30], which compensate for the reduced LCN2 formation by DMT1-deficient myeloid cells. However, we found evidence of cellular iron retention in infected DMT1fl/fl*^LysMCre^*^(+)^ mice, most strikingly by the observation of reduced FPN1 levels. Accordingly, strong tendencies towards increased splenic iron content and FRT levels were observed. Both are indicative for the impaired control of infection with intracellular bacteria [42,43]. The association of DMT1 functionality and FPN1 expression in vivo has been well established [20,21]. Our in vitro experiments revealed increased cellular iron content in DMT1-deficient BMDMs along with reduced expression of FPN1, FRT and TFR. When searching for an explanation for that phenomenon, we found reduced expression of iron chaperone PCBP2, a main intracellular iron shuttle between DMT1, FRT and FPN1 [44]. Further, PCBP2 functionality largely depends on the presence of DMT1 [45]. Notably, while iron stimuli strongly upregulated PCBP2 in controls^*LysMCre*(−)^, no induction was detected in DMT1-deficient macrophages. Here, we hypothesize that the disruption of DMT1 perturbs intracellular iron trafficking, consequently hindering distribution to essential iron-dependent targets. Ultimately, this might also explain the reduced expression of FPN1 and FRT upon the loss of DMT1. Consistently, NCOA4 levels were elevated, suggesting that DMT1fl/fl*^LysMCre^*^(+)^ macrophages aim at providing additional iron by degrading FRT. Of interest, decreased radioactive NTBI uptake of DMT1-deficient macrophages mainly excluded a relevant compensatory role of ZIP14. As NTBI and most likely TBI uptake, as reflected by radioactive iron uptake and TFR1 levels, appear to be reduced, the source of increased total iron content in DMT1-deficient macrophages needs to be addressed by further research. Shoe-Lin et al. have shown that DMT1 combined with NRAMP1 is necessary for efficient heme degradation, suggesting that elevated levels of heme are responsible for increased iron content [46]. Our data, however, reveal no alteration of heme-oxygenase 1 expression in DMT1-deficient macrophages (data not shown). Moreover, increased lysosomal iron accumulation due to DMT1 inhibition has been addressed by Turcu et al. [47]. Based on our data, we were not able to identify the exact localization of iron. However, calcein-quench assays do not measure endosomal or lysosomal iron under basal conditions [48]. Consequently, total intracellular iron of uninfected DMT1fl/flLysMCre^(+)^ BMDMs was increased, while the cytoplasmatic fraction was comparable between both genotypes (Figure 3d,e). This supports the hypothesis that iron must be localized within a calcein-impermeable compartment. In addition, Tenopoulou et al. demonstrate that oxidative stress results in phagolysosomal leakage and consequently the access of calcein to these compartments. Accordingly, infected BMDMs that lack DMT1 demonstrated increased labile iron (Figure 3d).

In conclusion, this study demonstrates the importance of DMT1 in nutritional immunity by controlling cellular iron homeostasis, affecting bacterial iron delivery and thus impairing intracellular bacterial proliferation.

## 4. Materials and Methods

### 4.1. Mice

DMT1fl/fl*^LysMCre^*^(+)^ and WT^*LysMCre*(−)^ C57BL/6J mice housed in the animal facilities of the Medical University of Innsbruck were kept under a constant light/dark cycle, fed with standard diet and had access to food and water ad libitum. The 8–12-week-old male mice were terminated by cervical dislocation to collect bone marrow. Animal handling was in accordance with approved guidelines from the Medical University of Innsbruck Animal ethics committee, and the Austrian Ministry for Science and Education (BMWFW-66.011/0091-WF/V/3b/2015).

### 4.2. Bone Marrow-Derived Macrophages and RAW264.7 Macrophage Culture

Isolation of BMDMs was performed by centrifugation of the dissected tibia and femur. Following lysis of erythrocytes (Ery Lysis Kit, R&D, Minneapolis, MN, USA), cells were washed several times with Dulbecco’s phosphate-buffered saline (PBS, PAA Laboratories, Toronto, ON, Canada). The left pellet was suspended in Dulbecco’s Modified Eagle’s Medium (DMEM, Lonza Ltd., Basel, Switzerland), supplemented with 10% fetal calf serum (FCS, Biochrome AG, Berlin/Heidelberg, Germany) and 2 mmol/L L-glutamine & PS (both obtained from PAA Laboratories), and seeded on 15 cm dishes. A macrophage colony-stimulating factor (M-CSF, Preprotech, Rocky Hill, NJ, USA) was added in dilution 1:2000 (25 ng/mL) to induce hematopoietic stem cell differentiation and proliferation. Cells were incubated at 37 °C in humidified air containing 5% carbon dioxide for six days and the medium was changed every other day. After 6 days, macrophages were washed twice (to remove antibiotics), scraped gently (Greiner, Frickenhausen, Germany) and suspended in antibiotica-free DMEM supplemented with 1% FCS and 2 mmol/L L-Glutamine. Next, cells were counted with an automatic cell counter (EVE^Tm^ cell counting obtained from NanoEntek, Hwaseong-si, Korea). For the read out of colon-forming units (CFU), RNA or flow cytometry analysis cells were seeded in 6-well plates (Falcon) at a density of 700,000 cells/mL For protein analysis macrophages were seeded on 10 cm^2^ dishes (Falcon, Copenhagen, Danish) at a density of 7,000,000 cells/dish. Macrophages were left untreated till the next day to adhere properly. RAW264.7 murine macrophage cells, obtained from American Type Culture Collection, were maintained and propagated in DMEM supplemented with 10% FCS.

### 4.3. Infection

*Salmonella* (ATCC 14028) were grown in Luria-Bertani broth (LB, Sigma-Aldrich, St. Louis, MO, USA) overnight. On the next day, 50 µL of overnight culture was suspended in 5 mL of LB medium. Bacteria were grown to the late-logarithmic phase, which is equivalent to an optic density of approximately 0.5 and were then quantified using a cell counter and analyzer (CASY, 45 μm capillary, OLS OMNI Life Science, Bremen, Germany). Macrophages were infected at a multiplicity of infection (MOI) of 10 for 1 h at 37 °C. Afterwards, macrophages were washed twice with PBS and repleted with DMEM containing 16 μg/mL Gentamicin sulphate solution (Carl Roth, Karlsruhe, Germany) to kill extracellular bacteria. Thereafter, macrophages were incubated for the indicated timepoints and subjected to further analysis.

### 4.4. CFU

To measure *Salmonella* proliferation, cells were washed three times with PBS (to eliminate gentamycin) and lysed with 0.5% Sodium Deoxycholate (Sigma-Aldrich) at indicated time points. The lysate containing recovered intracellular bacteria was plated in appropriate dilutions on LB-Agar plates (Sigma-Aldrich) and incubated for another 12 h at 37 °C. CFUs were counted manually the day after.

### 4.5. RNA Analysis

RNA extraction was performed by guanidine thiocyanate-phenol-chloroform extraction and analyzed as described previously [49].

### 4.6. Protein Analysis

Protein extracts from BMDMs were prepared using cytoplasmatic lysis buffer: Protein quantity was measured with Bradford Reagens (purchased from Biorad, Hercules, CA, USA), and 15–20 µg was loaded in 10% SDS-polyacrylamide gels. Proteins were separated by electrophoresis at 180 V for about 1 h and blotted on a PVDF-membrane (GE-Healthcare, Chicago, IL, USA) at 100 V. Protein bands were made visible with Posseau-Staining to cut aligned bands. Subsequently, antibody blocking in 5% milk powder prevented unspecific binding of antibodies. The first and secondary antibodies were applied including several washing steps in between TTPS. The protein quantity was evaluated using horse-radish peroxidase mediated chemilumenesce (Bio-Rad, 1:2000, anti-rabbit; 1:4000, anti-mouse; Dako, Glostrup, Denmark). The following antibodies were used: a mouse anti-TFR1 antibody (1:1000; Sigma Aldrich, MW: 100 kDa), a rabbit anti-LCN2 antibody (1:1000; Abcam, Cambridge, UK, MW: 23 kDa), a rabbit anti-FRT antibody (1:500; Sigma Aldrich, MW: 20 kDa), a rabbit anti-iNOS (1:1000; Abcam, Cambridge, UK, MW: 130 kDa), a rabbit anti-FPN1 antibody (1:2000; self-made, MW: 66 kDa), a rabbit anti-PCBP2 (1:1000, antikörper-online.de, 39 kDa) and a rabbit anti-ACTB antibody (1:500; Sigma Aldrich, MW: 45 kDa).

### 4.7. FACS Analysis

Flow cytometry was performed with a CytoFlex S™ Flow cytometer (Beckman Coulter, Brea, CA, USA). A Calcein quench assay (Calcein-AM, Thermo Fisher, Waltham, MA, USA) and ROS assays (CellRox, Thermo Fisher) were performed as described previously [50]. Briefly, BMDMs were stained/stimulated with two dyes: First, red fluorescent protein (RFP) expressing *Salmonella* (gracious gift from Dr. Dirk Bumann—Biozentrum Basel, Basel, Switzerland) were used to sort infected macrophages from uninfected ones. Second, either Calcein AcetoxyMethyl (Calcein-AM, ex/em 494/517 nm, Thermo Fisher) was used to measure the labile iron pool (LIP) and cell viability or CellRox (Green Reagent, ex/em 485/520 nm, ThermoFisher) and DAPI to measure oxidative stress and cell viability. Calcein-AM is non-fluorescent and membrane-permeable. After entering metabolically active phagocytes, cellular esterases hydrolyze acetoxymethyl to emit green fluorescence. The Calcein signal is quenched by labile iron. Therefore, a low mean fluorescence intensity (MFI) suggests high labile iron, whereas a high MFI indicates low labile iron [48].

### 4.8. ELISA

Lipocalin2 and Tumor necrosis factor α ELISA (both from R&D Systems, Minneapolis, MN, USA) IL6 and IL1b ELISA (BD Biosciences, San Jose, CA, USA) were used for the determination of these proteins in cell culture supernatants according to the manufacturer’s protocol.

### 4.9. Determination of Iron Uptake, Release and Content

For iron uptake and release experiments, cells were incubated for 2 h with 5 μM ^59^Fe-citrate, washed extensively and cultured in non-radioactive medium. To quantify iron import, macrophages were immediately harvested and radioactivity determined. For iron release assays, cells were incubated for an additional 4 h before the radioactivity of the culture supernatant was measured. For both assays, radioactivity was determined with a γ-counter (Perkin Elmer, Waltham, MA, USA), expressed as counts per minute (cpm) and normalized to the whole-cell lysate protein concentration to correct/account for cell density.

### 4.10. Tissue Iron Measurement

Tissue iron content was performed with acid-hydrolyzed tissue homogenates with a colorimetric method employing bathophenanthroline disulfonic acid and L-ascorbic acid in a sodium acetate assay. The calculated iron quantity was normalized to protein concentrations assessed by the Bradford method, for each sample.

### 4.11. In Vivo Methods

In vivo infection experiments were performed as described previously [51], Briefly, 500 CFUs of *Salmonella* were intraperitoneally administered to mice, and the experiments were terminated 72 h after infection. Bacterial load in the spleen and liver was determined either by plating serial dilutions of organ homogenates on LB agar (Sigma-Aldrich).

In each in vivo assay, surface body temperature was measured in at least 12-h intervals. The loss of reflexes (righting and grabbing reflex) and/or a body temperature drop of the animal of more than 5 °C compared with the pre-infection baseline were deemed a humane endpoint for infection and survival experiments. Mouse survival data were analyzed with a Cox regression and the Kaplan–Meier method using Wilcoxon’s test.

### 4.12. Statistics

Statistics were analyzed with Graphpad Prism version 8 for Windows (GraphPad Software, San Diego, CA, USA).

## Data Availability

Not applicable.

## Supplementary Materials

The following supporting information can be downloaded at: https://www.mdpi.com/article/10.3390/ijms23126789/s1.
