# Supplementary material for: DMT1 Protects Macrophages from Salmonella Infection by Controlling Cellular Iron Turnover and Lipocalin 2 Expression
Source: Int J Mol Sci. 2022 Jun 17;23(12):6789. doi: 10.3390/ijms23126789 (PMC9223531; doi:10.3390/ijms23126789)
Supplement: Supplementary file 1 [file ijms-23-06789-s001.zip › ijms-1733366-supplementary.pdf]

Supplemental Information

DMT1 protects macrophages from *Salmonella* infection by controlling cellular iron turnover and lipocalin 2 expression

Manuel Grander[1,2], Alexander Hoffmann[1,2], Markus Seifert[1,2], Egon Demetz[1], Philipp Grubwieser[1], Christa Pfeifhofer-Obermair[1], David Haschka[1*] and Günter Weiss[1,2 *]

1) Department of Internal Medicine II, and 2) Christian Doppler Laboratory for Iron metabolism and Anemia Research, Medical University of Innsbruck, 6020 Innsbruck, Austria;
mailto: manuel.grander@student.i-med.ac.at (M.G.); alexander.hoffmann@i-med.ac.at (A.H,); markus.seifert@i-med.ac.at (M.S.); egon.demetz@i-med.ac.at (E.D.); philipp.grubwieser@i-med.ac.at (P.G.); christa.pfeifhofer@i-med.ac.at (C.P.);
* Correspondence: david.haschka@i-med.ac.at or guenter.weiss@i-med.ac.at

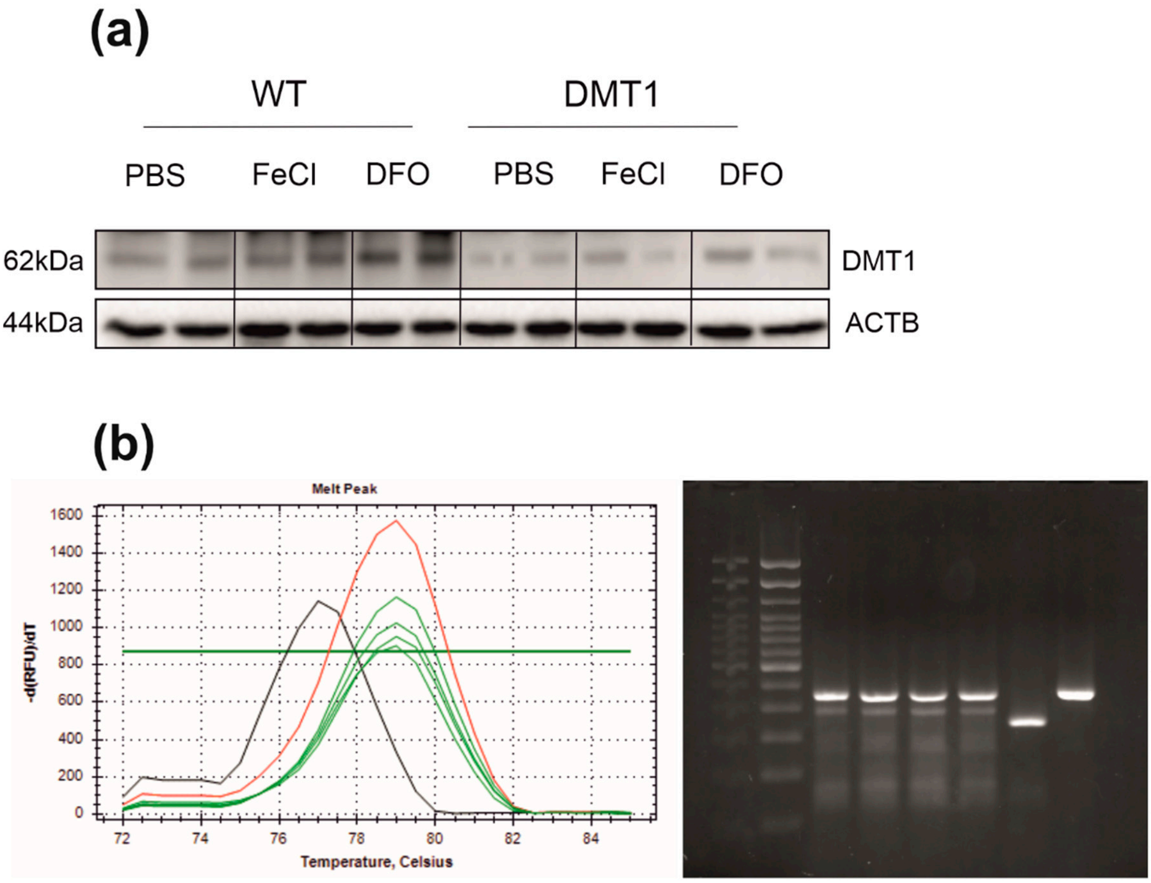

Figure S1. Ablation of macrophage DMT1 in DMT1fl/fl$^{LysMCre(+)}$ mice **(a)** DMT1fl/fl$^{LysMCre(+)}$and WT$^{LysMCre(-)}$ were stimulated with ferrous iron (FeCL), desferoxamin (DFO) or left untreated; **(b)** Ear punches from Cre transgenic mice as well as standard controls were subjected to PCR amplification using primers for DMT1 fl/fl as well as for a wildtype allele. Melt curves show peaks at 77 °C and 79 °C each, indicating specific binding temperature. Curve in red (DMT1) as well as black (WT) are standard control samples while curves in green indicate homozygously floxed DMT1 mice. **(b)**: Correspondent amplification products can be identified by size: Lane 1-4 indicate homozygously floxed PCR products at approximately 494 base pairs (floxed DMT1 alleles are longer thus contain more weight), Lane 5 indicates wildtype controls at approximately 393 bps and Lane 6 DMT1 fl/fl control. Size markers on the left.

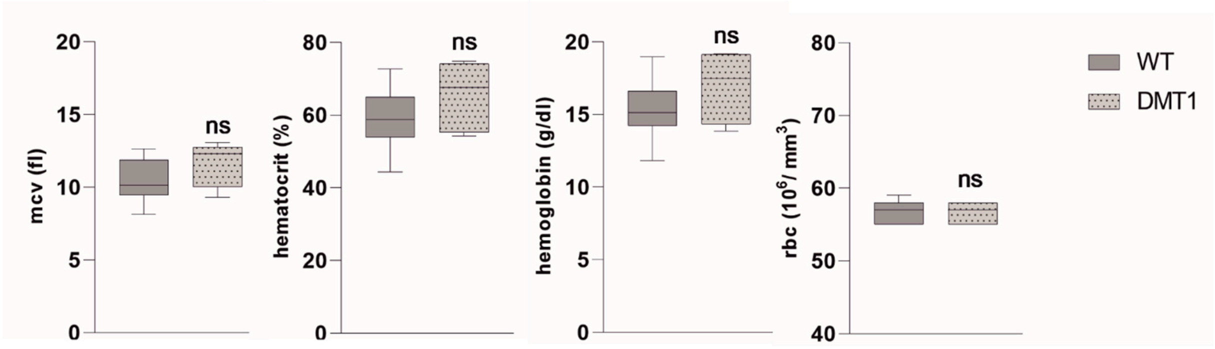

Figure S2. Macrophage DMT1 does not affect hematopoiesis Blood samples from from mice were compared by two-tailed unpaired t-test (two groups) or analysis of variance (ANOVA) using Bonferroni correction (more than two groups); n= mice per group. Box plots display whiskers with minimum to maximum. Statistical significancy $^*p < 0.05$, $^{**}p < 0.01$, $^{***}p < 0.001$ $^{****}p < 0.0001$, ns, no significance of differences; DMT1: DMT1 fl/fl $^{LyzMCre(+)}$, WT: wildtype.

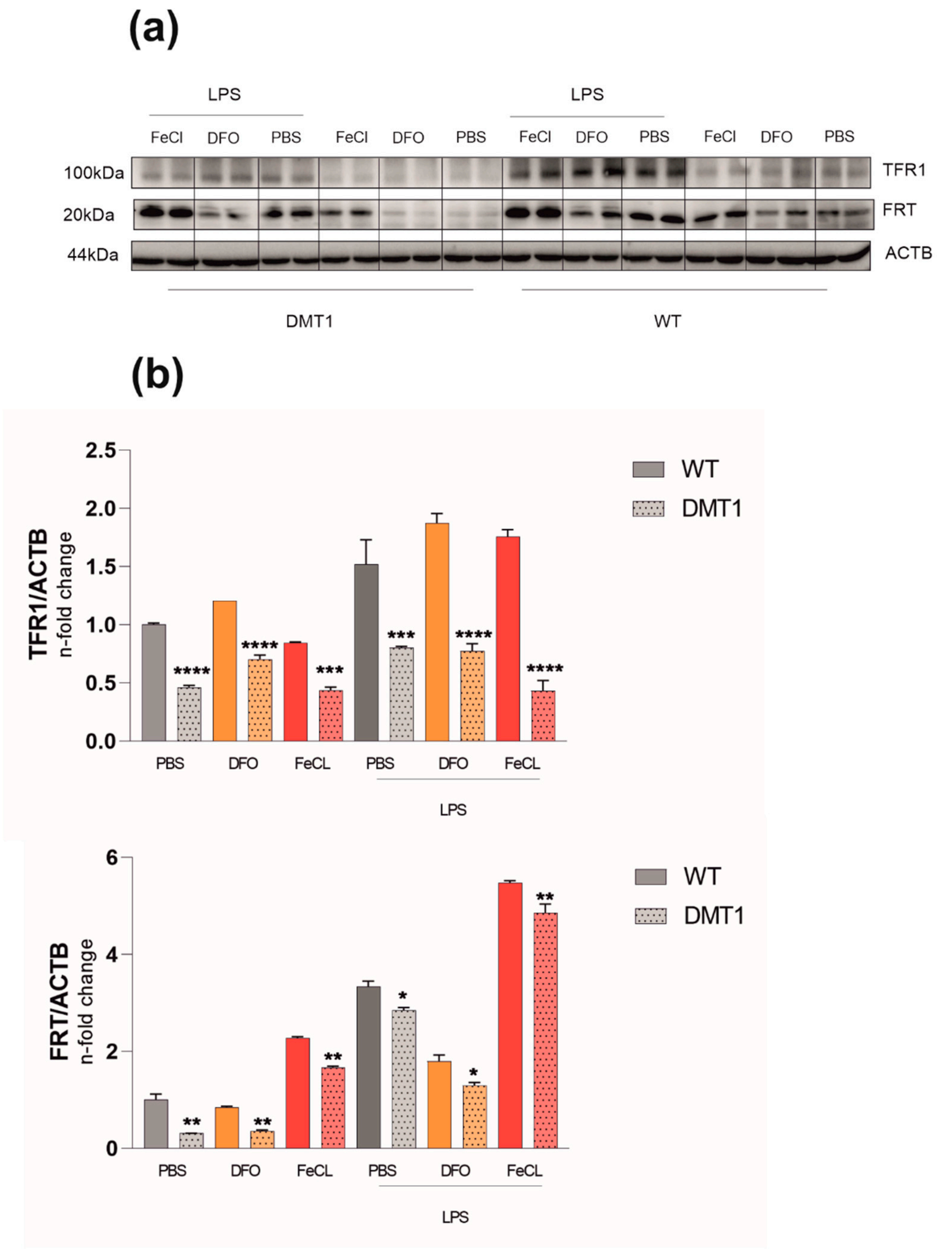

Figure S3. Aseptic inflammatory stimuli do not mitigate the observed phenotype (a) DMT1fl/fl$^{LyzMCre(+)}$ and WT$^{LyzMCre(-)}$ were stimulated with Lipopolysaccharide (LPS); **(b)** Protein levels of transferrin receptor 1 (TFR1), Ferritin (FRT) and βActin (ACTB) 6 hours poststimulationem (n=3). **(c)** Densitometric quantification of Western blot results; Duplicates or triplicates from at least two independent experiments were compared by two-tailed unpaired t-test (two groups) or analysis of variance (ANOVA) using Bonferroni correction (more than two groups); n= mice per group. Error bars expressed as SEM. Statistical significancy *$p < 0.05$, **$p < 0.01$, ***$p < 0.001$ ****$p < 0.0001$, ns, no significance of differences; DMT1: DMT1 fl/fl $^{LyzMCre(+)}$, WT: wildtype.

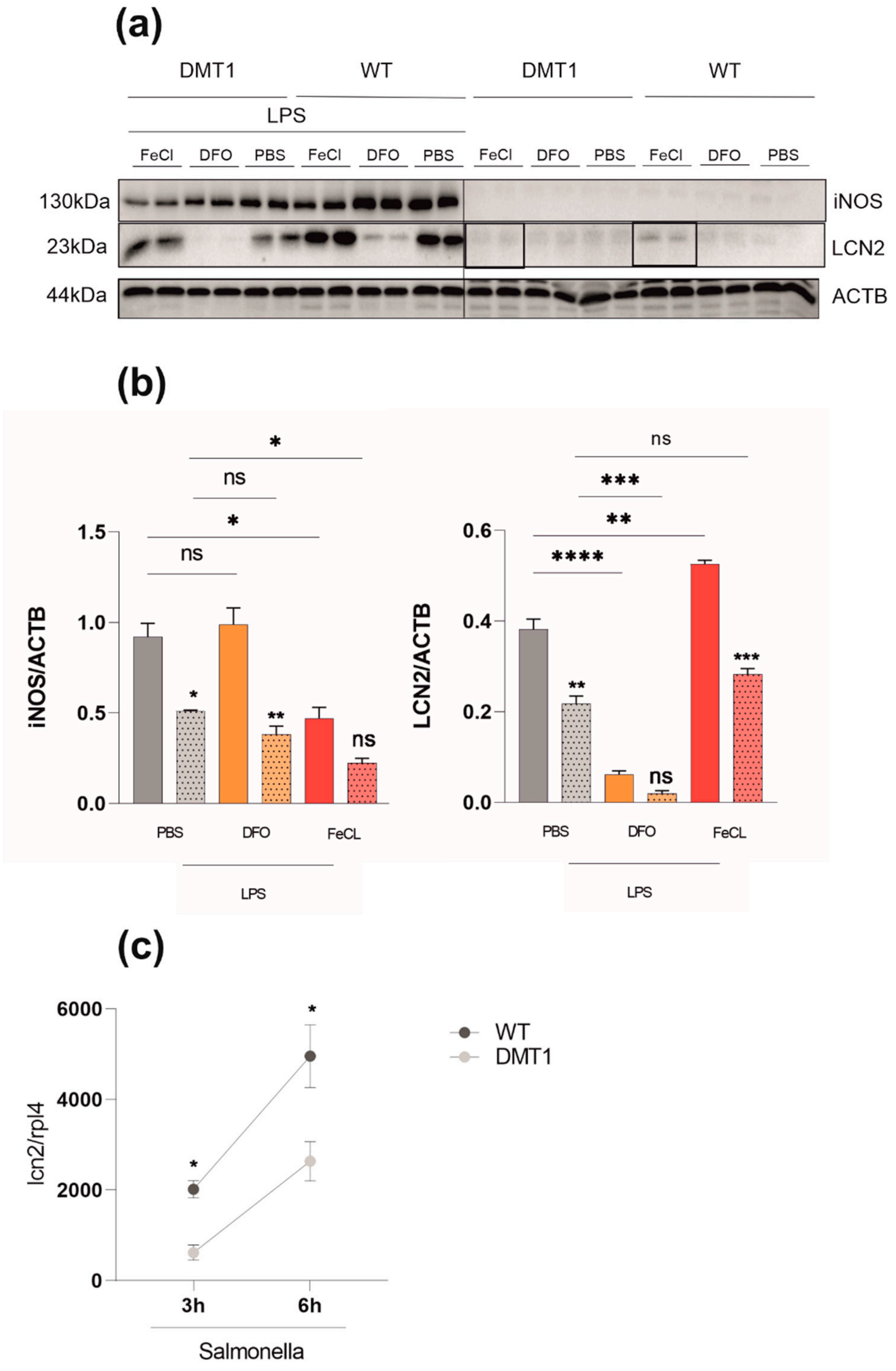

Figure S4. LCN2 is regulated by inflammation and iron fluctations (a) DMT1fl/fl $^{LyzMCre(+)}$ and WT$^{LyzMCre(-)}$ were stimulated with Lipopolysaccharide (LPS) overnight; **(b)** Protein levels of transferrin receptor 1 (TFR1), Ferritin and βActin 6 hours poststimulationem (n=3). Densiometric quantification of Western blot results **(c)** Relative mRNA expression (n=3) of genes involved in iron transport: Ribosomal Protein L4 (Rpl4) was used as a reference gene (averages ± SEM); n= mice per group. Error bars expressed as SEM. Statistical significancy *p < 0.05, **p < 0.01, ***p < 0.001 ****p < 0.0001, ns, no significance of differences; DMT1: DMT1 fl/fl $^{LyzMCre(+)}$, WT: wildtype.

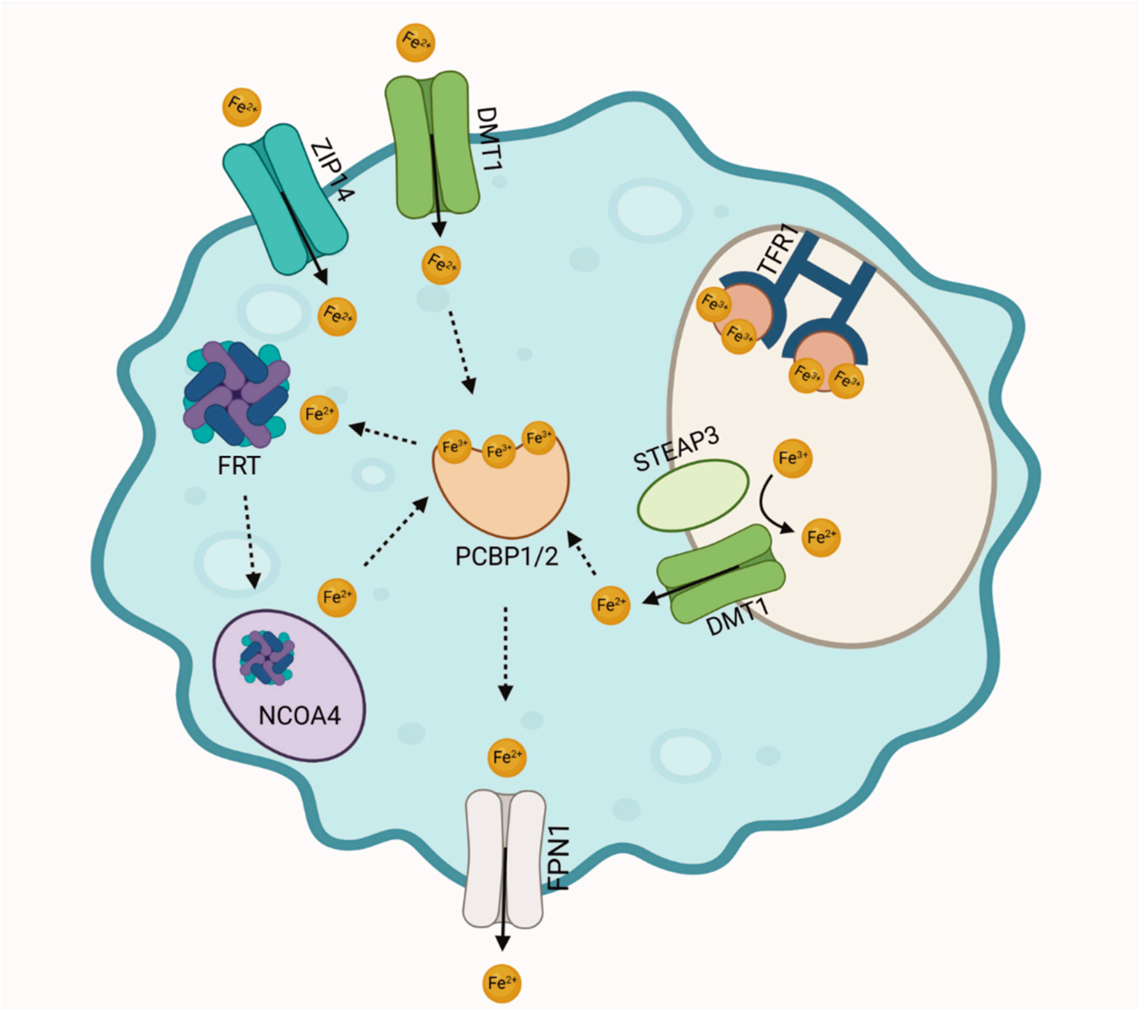

Figure S5. Iron metabolism in macrophages: DMT1 and ZIP14 mediate transfer of non-transferrin bound iron from extracellular into intracellular space. Importantly, DMT1 transports ferrous iron through direct protein-protein interaction with the iron chaperone PCBP1/2. As a next step, PCBP1/2 trafficks iron to its desired targets such as the iron storage protein FRT or to the iron exporter FPN1. In addition, DMT1 mediates transferrin bound iron uptake via extraction of iron from the endosome. As DMT1 transfers only bivalent metals, the ferrireductase STEAP3 is necessary to reduce ferric iron bound to transferrin. The enzyme NCOA4 is necessary to degradate FRT and thus to mobilize iron. Illustration created with Biorender.com.
